# E-钙粘蛋白复合体与肺癌的侵袭转移

**DOI:** 10.3779/j.issn.1009-3419.2010.03.11

**Published:** 2010-03-20

**Affiliations:** 1 610041 成都，四川大学华西临床医学院，华西医院胸心外科 Department of Thoracocardiac Surgery, West China Hospital, Sichuan University, Chengdu 610041, China; 2 300052 天津，天津医科大学总医院，天津市肺癌研究所，天津市肺癌转移与肿瘤微环境重点实验室 Tianjin Key Laboratory of Lung Cancer Metastasis and Tumor Microenviroment, Tianjin Lung Cancer Institute, Tianjin Medical

肺癌是发病率和死亡率增长最快、对人类健康和生命威胁最大的恶性肿瘤。肿瘤的侵袭转移是其恶性特征和标志，也是肺癌患者治疗失败和死亡的主要原因。肿瘤细胞从原发部位脱落、迁移是肿瘤转移的重要环节，这一过程与肿瘤细胞间粘附功能的降低密切相关。由上皮型钙粘蛋白（E-cadherin）与其胞内域相连接的β-连环蛋白（β-catenin）构成的E-钙粘蛋白-连环蛋白复合体（E-cadherin/catenin）是细胞间粘附分子的重要部分，在抑制肺癌的侵袭转移过程中发挥着极其重要的作用。本文就E-钙粘蛋白复合体的构成及其与肺癌侵袭转移的关系做一综述。

## 1 E-钙粘蛋白复合体的构成

E-钙粘蛋白是一种介导同质细胞间相互粘附的Ca^2+^依赖性跨膜糖蛋白，主要分布于上皮组织中，其基因*CDH1*定位于染色体16q22.1，cDNA全长48 kb，在胞质内先合成135 kDa的蛋白前体，经剪切修饰成熟后转移至细胞膜发挥其生物等功能。E-钙粘蛋白的胞外区是由5个串联重复的结构单元构成，每个结构单元大约含有110个氨基酸残基，相邻的重复结构单元之间是Ca^2+^的结合位点，其中第一个重复序列（最外侧）包含一个“组-丙-缬”（HAV）三肽的模体，它决定着E-钙粘蛋白与同质细胞间的特异性识别和相互作用。跨膜区由32个氨基酸组成的疏水结构域。E-钙粘蛋白的胞内域含有SH1和SH2区：SH1区可以和β-连环蛋白（γ-连环蛋白）直接结合；SH2区和p120ctn结合，发挥调节粘附分子二聚体聚集和解离的作用（图 1）。

**1:**
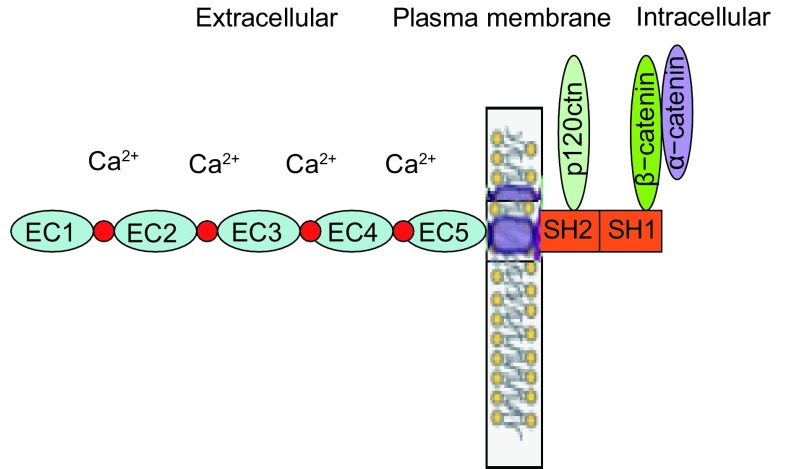
E-钙粘蛋白的结构和功能域 The structure and function domains of E-cadherin

β-连环蛋白是一种胞内可溶性的多功能蛋白，编码β-catenin的基因为*CTNNB1*，定位于染色体3p21上，全长为23.2 kb，由781个氨基酸组成，分子量约为92 kDa。肽链的中部有12个-14个各由42个氨基酸残基组成的重复序列（称arm区域），该区域可以和多种蛋白受体结合而发挥功能。β-连环蛋白在细胞内存在有两种形式：①游离型：主要存在于细胞浆中，通过与大肠腺瘤样息肉蛋白（adenomatous polyposis coli, APC）、糖原合成酶激酶-3β（glycogen synthase kinase-3β, GSK-3β）和轴蛋白（axin）形成的“破坏复合物”结合后被GSK-3β和酪蛋白激酶1α（casein kinase 1, CK1α）磷酸化，进而泛素化被蛋白水解酶降解，从而使其在细胞浆中维持一个较低的水平；②结合型：β-连环蛋白由胞浆转移至细胞膜下和E-钙粘蛋白形成复合体，参与细胞之间的粘附，进入细胞核的β-连环蛋白与T细胞因子/淋巴细胞增强因子（T cell factor/lymphoid enhancer factor, Tcf/LEF）结合参与细胞内基因的表达，引起细胞的增殖，但磷酸化的β-连环蛋白进入细胞核后，虽能与TcF/LEF相互作用，却不能和DNA结合，无法启动相应靶基因的转录^[1]^（图 2）。

**2:**
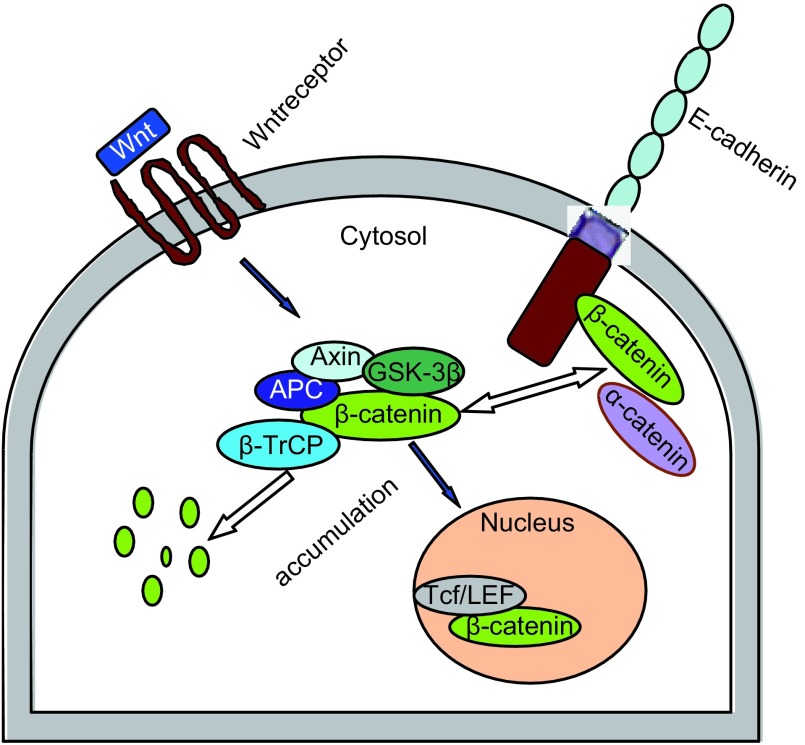
β-连环蛋白在细胞内的功能和分布 The function and distribution of β-catenin in the cell

E-钙粘蛋白复合体的形成过程是在E-钙粘蛋白穿膜时开始的，首先β-连环蛋白（γ-连环蛋白）和E-钙粘蛋白的胞内肽段结合，然后在胞膜上α-连环蛋白与β-连环蛋白（γ-连环蛋白）的氨基端结合而将E-钙粘蛋白复合体锚定在肌动蛋白细胞骨架上。细胞膜上与E-钙粘蛋白结合的β-连环蛋白可以和细胞浆内的β-连环蛋白相互交换，这种交换形成一种平衡状态，并由此调节同质细胞之间的粘附作用。

## 2 E-钙粘蛋白复合体在肺癌侵袭转移中的作用

E-钙粘蛋白是参与细胞之间粘附连接的主要分子，发挥着维持细胞极性和组织结构完整性的功能，它的抑癌作用主要是通过与β-连环蛋白形成复合体介导同质细胞之间的相互粘附，抑制肿瘤细胞的迁移和侵袭性，同时E-钙粘蛋白竞争性和β-连环蛋白结合，降低细胞内游离型β-连环蛋白水平，从而抑制其参与Wnt信号通路细胞增殖基因的表达^[2-4]^。E-钙粘蛋白复合体中无论钙粘蛋白本身或其组分的异常变化都可能引起细胞之间的粘附能力减弱以及肿瘤细胞的运动能力增强，从而导致细胞容易脱离、侵袭和转移^[5]^。

近年来的研究发现，在肺癌的侵袭与转移过程中E-钙粘蛋白复合体功能异常可能通过以下几个环节参与肺癌侵袭转移的调控。

### 2.1 基因突变

基因突变是引起蛋白表达水平改变和功能障碍的主要机制之一，在多种恶性肿瘤的研究^[6]^中发现，E-钙粘蛋白基因的点突变、缺失均可导致E-钙粘蛋白表达水平的下调和功能结合区域的改变，从而引起细胞-细胞之间连接疏松。陈晓峰等^[7]^应用聚合酶链反应和单链构象多态性分析技术对53例非小细胞肺癌（non-small cell lung cancer, NSCLC）原发灶组织和5例肺部其他良恶性病变组织中的E-钙粘蛋白基因突变情况进行检测，结果显示仅3例NSCLC发生E-钙粘蛋白基因突变，E-钙粘蛋白基因的突变率在肺癌和非肺癌之间无统计学差异。目前，在肺癌组织细胞中有关E-钙粘蛋白基因突变的文献报道较少，且突变发生率亦较低，据此推测E-钙粘蛋白基因突变在肺癌的侵袭转移过程中可能不是主要事件。

有研究显示β-连环蛋白基因的变异可以使细胞之间的粘附能力下降。当前对β-连环蛋白基因突变的研究主要集中于基因外显子3（外显子3的第33、37、41、45位密码子编码区域，构成连环蛋白氨基末端糖原合成酶激酶-3β和酪蛋白激酶1α的磷酸化位点，是β-连环蛋白正常被磷酸化后降解维持细胞内低水平状态所必需的），当其出现缺失或变异时不仅影响其参与的粘附功能，同时会导致β-连环蛋白与GSK-3β结合障碍而不能被降解，在细胞内异常积累并转移至细胞核，激活异常的Wnt信号通路，从而促进细胞增殖^[8]^。李利亚等^[9]^应用PCR-SSCP和DNA测序的方法对36例NSCLC术后标本进行β-连环蛋白外显子3的突变分析，结果显示β-连环蛋白基因外显子3有突变，在我国其突变率较国外报道高；并且β-连环蛋白基因外显子3的突变与NSCLC的淋巴转移和肿瘤的分化程度相关，提示β-连环蛋白基因外显子3的突变可能与NSCLC转移密切相关。

### 2.2 甲基化

CpG岛异常甲基化是肿瘤抑制基因失活的主要机制之一，CpG岛高度甲基化可能改变染色体局部构象，从而阻止转录因子与相应启动子的结合来抑制转录过程，E-钙粘蛋白启动子区域的CpG岛异常高度甲基化会导致*CDH1*基因转录失活而引起E-钙粘蛋白表达的下调。一般认为启动子甲基化引起的E-钙粘蛋白表达下调是动态变化的，原发肿瘤发生侵袭时启动子区发生高度甲基化，此时E-钙粘蛋白表达下降；当侵袭转移的肿瘤细胞到达一个适合其定居的环境后，启动子甲基化水平可显著下降，E-钙粘蛋白表达水平可恢复，便于细胞粘附形成转移灶。Wang等^[10]^对95例NSCLC肿瘤组织标本检测发现*CDH1*基因在肿瘤组织中出现甲基化的频率比相应肿瘤周围组织显著增高，同时检测*CDH1*基因的甲基化程度与E-钙粘蛋白表达水平的降低相关。王红兵等^[11]^应用甲基化特异性PCR技术检测22例肺癌组织、相应的癌旁组织和9例正常肺组织中E-钙粘蛋白基因启动子CpG岛甲基化水平，结果显示肺癌中E-钙粘蛋白基因启动子CpG岛完全甲基化率为13.6%（3/22），部分甲基化率为27.3%（6/22），总甲基化率为40.9%（9/22），显著高于相应癌旁组织中该基因的甲基化率[9.1%（2/22）]；9例正常肺组织中该基因未发生甲基化，提示E-钙粘蛋白启动子异常甲基化可能是肺癌侵袭转移中的常见事件。亦有较多文献^[12-14]^报道E-钙粘蛋白基因甲基化水平可作为评估肺癌浸润转移和预后的有效指标。

### 2.3 E-钙粘蛋白基因转录抑制

基因的转录抑制是E-钙粘蛋白表达下调的重要机制。人E-钙粘蛋白启动子区域存在的E-box含有保守的5′-CACCTG序列，该序列为helixloop-helix（HLH）转录因子家族的结合位点。在上皮肿瘤细胞中，当E-钙粘蛋白基因启动子的E-box结合转录抑制因子后可下调E-钙粘蛋白的表达。这些转录抑制因子有Slug^[15]^、Twist^[16]^、Snail和ZEB^[17]^等，其中Snail和ZEB因子与羧基末端连接蛋白（C-terminal-binding proteins, CtBPs）结合后可以恢复去乙酰化酶活性，使染色质核小体的构象处于紧缩状态，因此不能和DNA结合而抑制E-钙粘蛋白基因的表达。此外，这些转录抑制因子和*CDH1*基因的启动子结合，能够诱导上皮细胞向间质细胞表型的转化（epithelial-mesenchymal transition, EMT），同时伴随E-cadherin向N-cadherin转化，使肿瘤细胞获得侵袭的能力^[18]^。

### 2.4 磷酸化和糖基化

蛋白分子晶体结构分析显示：β-连环蛋白中心区域由12个arm重复结构形成一个超螺旋的环，形成一个带正电的沟，能结合带负电的钙粘蛋白受体、降解复合体以及LEF/TEF转录因子。因此，当E-钙粘蛋白和β-连环蛋白发生异常磷酸化时将改变分子的带电性和（或）分子的空间构象，影响复合体的正常形成和功能。β-连环蛋白的酪氨酸磷酸化可引起β-连环蛋白和E-钙粘蛋白分子之间相互作用的破坏，致使细胞内游离的β-连环蛋白水平增高^[19-20]^，而β-catenin被丝氨酸/苏氨酸磷酸化后能够增强β-连环蛋白和E-钙粘蛋白的相互作用^[21]^，进一步研究发现：β-连环蛋白的第654位酪氨酸磷酸化能降低β-连环蛋白和E-钙粘蛋白的结合力而促进β-连环蛋白和TATA连接蛋白的协同作用^[22]^，β-连环蛋白的第142位酪氨酸磷酸化则能降低β-连环蛋白和α-连环蛋白的相互作用^[23]^。β-连环蛋白的功能和酪氨酸激酶的活性有密切关系，酪氨酸激酶的激活能够增强细胞核内β-连环蛋白的信号^[24]^，相反酪氨酸激酶的失活能够增强β-连环蛋白和E-钙粘蛋白的结合而相应降低TCF/β-catenin调节的基因转录——在酪氨酸磷酸化的β-连环蛋白经去磷酸化药物（geldanamycin）处理后，酪氨酸磷酸化的水平降低并促进它和钙粘蛋白的相互作用，致使细胞运动能力的降低^[25-26]^。对于E-钙粘蛋白，有研究^[27-28]^显示当其胞浆域被GSK-3β或CK-Ⅱ磷酸化时，可调节E-钙粘蛋白和β-连环蛋白之间的亲合力，最终增强细胞之间的粘附作用，可能与其带电荷的改变有关。另外，文献^[29-30]^报道E-钙粘蛋白糖基化后可以改变细胞之间的粘附作用，E-钙粘蛋白介导的细胞之间的粘附能力在其糖基化降低时增强而糖基化升高时减弱，结构模型显示E-钙粘蛋白核心岩藻糖基化（core fucosylation）可以改变E-钙粘蛋白的三维构象，其介导的细胞间粘附功能受到抑制，使肺癌细胞的转移性增高。

### 2.5 细胞内异常分布

人们通常认为E-钙粘蛋白表达升高会降低肿瘤细胞的侵袭转移力，相反E-钙粘蛋白表达下降会导致转移潜能的提高，并得到实验证实。但有报道^[31-34]^认为，E-钙粘蛋白表达较高的肿瘤仍具有较高的侵袭转移能力，可能是由于E-钙粘蛋白不能正常定位于细胞膜上，从而丧失粘附功能。细胞内异常分布可能是其中一个原因，亦有较多肿瘤细胞内两者存在异常分布的报道。有研究^[35]^发现，在人支气管肺癌中，E-钙粘蛋白在细胞质内的重新分布可能与E-钙粘蛋白酪氨酸磷酸化水平下调有关，但具体调节机制目前尚不清楚。β-连环蛋白在细胞膜和细胞核具有不同的生物功能，应用免疫组化方法对多数恶性肿瘤的研究结果大都显示β-连环蛋白的细胞内异常分布：细胞膜上分布减少，出现核内的聚集^[36, 37]^。在肺癌组织细胞中亦出现β-连环蛋白空间分布的异常^[38]^。

### 2.6 其他影响因素

p120-catenin是E-钙粘蛋白复合体的一个重要调节蛋白，在和E-钙粘蛋白结合时可以稳定E-钙粘蛋白在细胞膜上的稳定性，当其和E-钙粘蛋白解离后，E-钙粘蛋白则易被内吞降解^[34, 39, 40]^。另外研究发现，*nm23-H1*基因在E-钙粘蛋白和β-连环蛋白的表达调控中起重要作用，将*nm23-H1*基因转染肺癌细胞株L9981后能明显上调E-钙粘蛋白和β-连环蛋白基因和蛋白的表达水平^[41-43]^，而应用siRNA干扰技术对肺癌细胞NL9980中的*nm23-H1*基因进行沉默后E-钙粘蛋白基因的表达水平明显下调^[44]^。

## 3 E-钙粘蛋白复合体在肺癌中的异常表达与肺癌临床特征的关系

大量的临床研究^[45, 46]^显示E-钙粘蛋白和β-连环蛋白蛋白在NSCLC中有不同程度的分布异常和表达下调。Xu等^[47]^应用免疫组化的方法检测了100例NSCLC中β-连环蛋白的表达，结果显示80%的病例出现膜上表达的降低，26%的病例出现细胞核内异常表达。Kase等^[48]^对331例肺癌组织中E-钙粘蛋白和β-连环蛋白的表达进行分析，42%（138/331）的病例出现E-钙粘蛋白的降低，37%（122/331）的病例出现β-连环蛋白表达降低。

许多研究已证明：E-钙粘蛋白复合体的表达水平与肺癌的组织类型、分化程度及淋巴结转移有关。唐小军等^[49]^的研究显示肺鳞癌组织中E-钙粘蛋白、β-连环蛋白的表达水平显著低于肺腺癌和腺鳞癌，而腺癌与腺鳞癌之间无明显差异；低分化肺癌中E-钙粘蛋白的表达低于中-高分化者，而β-连环蛋白的表达水平与分化程度没有相关性；有淋巴结转移（N1-3）以及Ⅲ、Ⅳ期肺癌中的表达水平均分别低于无淋巴结（N0）和Ⅰ、Ⅱ期肺癌（*P* < 0.01）。Choi等^[50]^应用组织微阵和免疫组化方法对141例术后Ⅰ期肺癌标本中E-钙粘蛋白和β-连环蛋白的表达水平分析发现，E-钙粘蛋白和β-连环蛋白缺失或低表达分别达60%和45%，且鳞癌中的异常表达（72.5%）明显高于腺癌（36.6%），两者之间有明显差异。Nozawa等^[51]^应用免疫组化的方法对35例肺癌组织中E-钙粘蛋白复合体的表达进行分析，结果显示复合体在膜上表达水平的降低与肺癌的低分化有明显相关性，而与淋巴结转移没有统计意义的相关性。然而也有研究^[52-54]^显示有淋巴结转移的癌组织E-钙粘蛋白、β-连环蛋白的表达明显低于无淋巴结转移组。E-钙粘蛋白复合体在肺癌淋巴结转移相关性的研究中出现的不同结果可能与研究方法、样本量的大小及肿瘤的组织类型不同有密切关系。许多研究已证明：E-钙粘蛋白复合体的表达水平与肺癌的组织类型、分化程度及淋巴结转移有关。唐小军等^[49]^的研究显示肺鳞癌组织中E-钙粘蛋白、β-连环蛋白的表达水平显著低于肺腺癌和腺鳞癌，而腺癌与腺鳞癌之间无明显差异；低分化肺癌中E-钙粘蛋白的表达低于中-高分化者，而β-连环蛋白的表达水平与分化程度没有相关性；有淋巴结转移（N1-3）以及Ⅲ、Ⅳ期肺癌中的表达水平均分别低于无淋巴结（N0）和Ⅰ、Ⅱ期肺癌（*P* < 0.01）。Choi等^[50]^应用组织微阵和免疫组化方法对141例术后Ⅰ期肺癌标本中E-钙粘蛋白和β-连环蛋白的表达水平分析发现，E-钙粘蛋白和β-连环蛋白缺失或低表达分别达60%和45%，且鳞癌中的异常表达（72.5%）明显高于腺癌（36.6%），两者之间有明显差异。Nozawa等^[51]^应用免疫组化的方法对35例肺癌组织中E-钙粘蛋白复合体的表达进行分析，结果显示复合体在膜上表达水平的降低与肺癌的低分化有明显相关性，而与淋巴结转移没有统计意义的相关性。然而也有研究^[52-54]^显示有淋巴结转移的癌组织E-钙粘蛋白、β-连环蛋白的表达明显低于无淋巴结转移组。E-钙粘蛋白复合体在肺癌淋巴结转移相关性的研究中出现的不同结果可能与研究方法、样本量的大小及肿瘤的组织类型不同有密切关系。

E-钙粘蛋白及其相关的β-连环蛋白在肺癌的预后方面已进行了广泛的研究，E-钙粘蛋白复合体的异常表达和不良预后成正相关^[46, 55]^，E-钙粘蛋白的异常是可以作为肺癌侵袭转移和不良预后的一个独立指标，多因素分析显示连环蛋白表达水平低的患者生存期短^[51]^，免疫组化预后分析亦表明β-连环蛋白的低表达和不良预后相关^[47, 50-54]^。

## 4 总结

综上所述，E-钙粘蛋白复合体表达下调或/和功能丧失与肺癌的分化、侵袭转移和预后密切相关。E-钙粘蛋白复合体在细胞膜上的形成和调控涉及到多种机制，尤其是β-连环蛋白结合型和游离型之间平衡状态的调控，直接影响到其对肿瘤细胞的生物学作用，但这种复杂的调节机制目前还不甚清楚，仍需进一步的研究。最近研究^[56]^报道，吲哚美辛成浓度和时间依赖性地增加结肠癌细胞内E-钙粘蛋白的表达，并使β-连环蛋白从细胞浆和细胞核内转位到细胞膜上，增强细胞之间的粘附作用，这提示可以通过纠正E-钙粘蛋白和β-连环蛋白的表达和分布异常以增强细胞之间粘附作用的途径来降低肿瘤细胞的侵袭转移潜能，这也可能成为肿瘤治疗的一个新靶点。因此通过对E-钙粘蛋白复合体调控机制的进一步深入研究，将对阐明肺癌的侵袭转移有着重要的意义，也将对肺癌的靶向治疗提供一个新的思路。
